# Hemoptysis secondary to pulmonary vein stenosis after radiofrequency ablation for atrial fibrillation: A case report and literature review

**DOI:** 10.1016/j.jimed.2020.03.008

**Published:** 2020-03-30

**Authors:** Zhiming Xuan, Boyu Liu, Minjun Ci, Zhe Wang, Yong Fan

**Affiliations:** a Tianjin Medical University, Tianjin, Guangdong Road 1^#^, Hexi District, 300202, Tianjin, China; b Department of Radiology, Tianjin Medical University General Hospital, Anshan Road 154^#^, Heping District, 300052, Tianjin, China

**Keywords:** Hemoptysis, Pulmonary vein, Radiofrequency ablation, Atrial fibrillation

## Abstract

**Objectives:**

Pulmonary vein stenosis (PVS) is a known complication after radiofrequency ablation of atrial fibrillation (RAAF) and is often misdiagnosed owing to lack of awareness regarding PVS among noncardiologists. Misdiagnosis results in unnecessary treatment; therefore, greater understanding of PVS can improve the management of these patients.

**Methods:**

We report the case of a 38-year-old man with a history of RAAF who presented with massive hemoptysis. His symptoms persisted despite undergoing transcatheter bronchial artery embolization on two occasions.

**Results:**

Pulmonary computed tomography angiography revealed a completely occluded left superior pulmonary vein. Considering the patient’s history of RAAF, we diagnosed him with RAAF-induced PVS and performed left superior lobectomy after which hemoptysis did not recur.

**Conclusions:**

Unexplained massive hemoptysis should alert clinicians regarding the possibility of RAAF-induced PVS. Balloon angioplasty and stent placement are used to treat PVS; however, their efficacy is controversial considering the high recurrence rates associated with these interventions.

## Introduction

Hemoptysis is an important clinical manifestation of respiratory diseases. Common causes of hemoptysis observed in clinical practice include bronchitis, bronchogenic carcinoma, bronchiectasis, infections, and tuberculosis.^1^ Uncommon causes of hemoptysis include myofibroblastoma, hemangioma, angiosarcoma, hamartoma, Dieulafoy’s disease of the bronchus, bronchial artery-pulmonary artery malformations, and pulmonary vein stenosis (PVS).^2^, ^3^, ^4^, ^5^, ^6^ We report a rare case of a 38-year-old man who presented with recurrent hemoptysis secondary to PVS after undergoing radiofrequency ablation for atrial fibrillation (RAAF).

## Case report

**Fig. 1:**
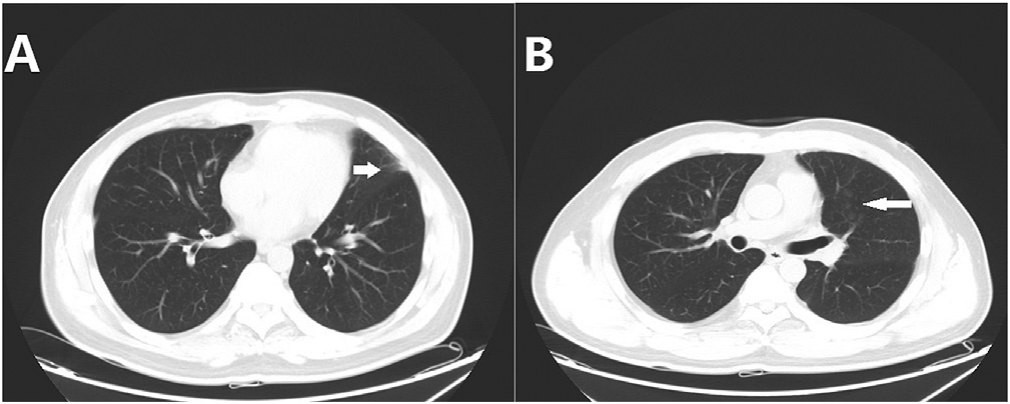
Pulmonary CT scan obtained before the procedure showing scattered ground-glass opacities in the left upper lobe (arrow), suggesting alveolar hemorrhage (A); A high-density stripe in the left upper lobe (arrow), suggesting chronic inflammation (B). CT: computed tomography.

**Fig. 2:**
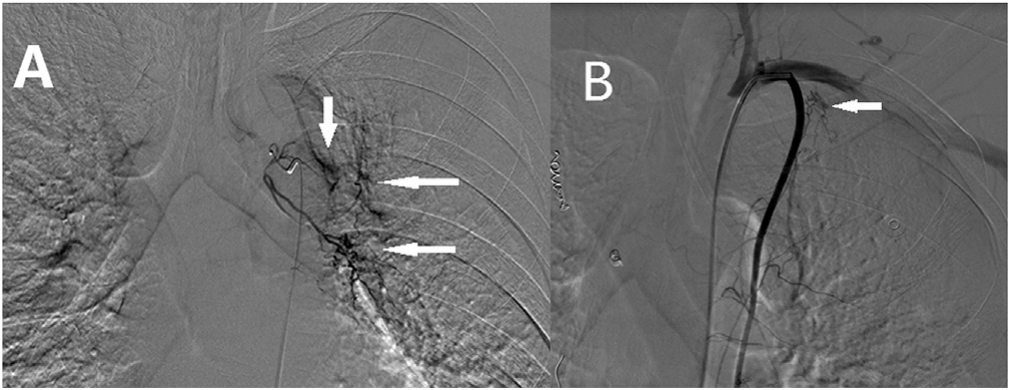
Left bronchial artery angiography scan showing an enlarged bronchial artery with an extensive branching pattern, and contrast agent extravasation (arrows) is observed in the left upper lung field (A); an ectopic bronchial artery (arrow) originating from the left internal thoracic artery is observed to be the source of hemorrhage (B).

The patient was referred to the emergency department at our hospital because of massive hemoptysis. He reported a history of catheter ablation performed for atrial fibrillation, 10 days prior to presentation. Chest radiography did not reveal any positive findings. Thoracic computed tomography (CT) scan revealed scattered ground-glass opacities in the left upper lobe, suggesting alveolar hemorrhage **(**Fig. 1A**)**. Additionally, a high-density stripe observed in the left upper lobe suggested chronic inflammation **(**Fig. 1B**)**. Medical treatment with vasopressin and hemagglutinin was ineffective. Left bronchial artery angiography revealed an enlarged bronchial artery showing an extensive branching pattern, and contrast agent extravasation was observed in the left upper lung field **(**Fig. 2A**)**. We performed transcatheter bronchial artery embolization using polyvinyl alcohol embolization particles (Cook Inc., IN, USA), gelatin sponge particle embolic agent (Hangzhou Alicon Pharmaceutical Sci & Tec Co. Ltd., Hangzhou, China) and fibered platinum coils (Boston Scientific, MA, USA). Hemoptysis was controlled immediately after the procedure; however, massive hemoptysis recurred on the second day. Repeat angiography revealed that an ectopic bronchial artery originating from the left internal thoracic artery was the source of hemorrhage **(**Fig. 2B**)**; therefore, we performed transcatheter embolization of the ectopic bronchial artery. Unfortunately, hemoptysis was controlled only for 2 days after the procedure and recurred thereafter.

**Fig. 3:**
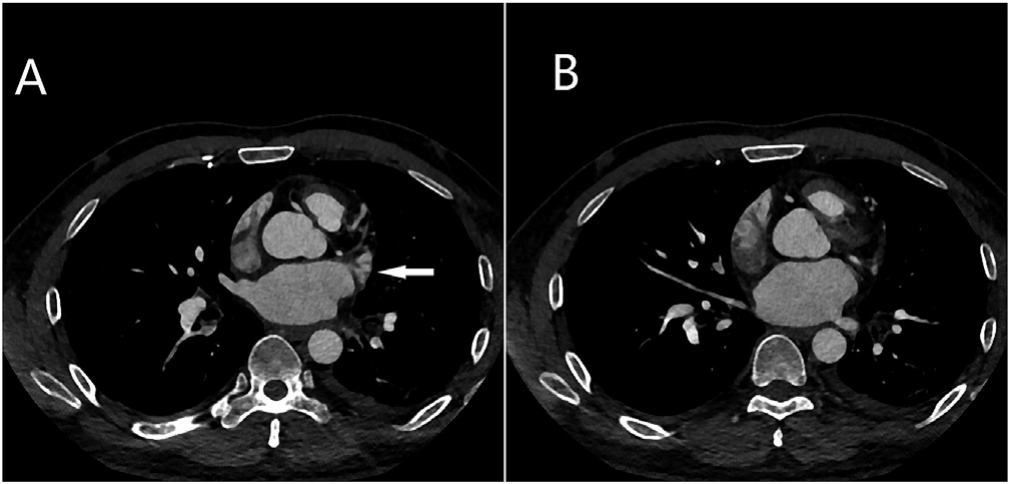
Pulmonary vein CT angiography scan showing multiple filling defects at the proximal end of the left superior pulmonary vein, representing thrombosis (A); The distal trunk and branches of the left superior pulmonary vein are completely occluded (B). CT: computed tomography.

Pulmonary CT angiography was performed to detect potential ectopic bronchial arteries, and complete occlusion of the left superior pulmonary vein was accidently discovered during this examination **(**Fig. 3A and B**)**. Based on his history of RAAF, hemoptysis was attributed to RAAF-induced PVS in this patient. The patient underwent left superior lobectomy after which hemoptysis did not recur.

## Discussion

Since it was first reported in 1998,^7^ radiofrequency ablation (RAF) is used as an effective therapeutic option for recurrent symptomatic paroxysmal atrial fibrillation or persistent symptomatic atrial fibrillation in patients refractory to antiarrhythmic medications.^8^ Notably, the widespread application of RAF in clinical practice has led to a high risk of PVS in these patients. Reportedly, PVS is known to have occurred in 1%–42% of patients undergoing RAF.^9^^,^^10^ RAAF is a relatively new procedure; therefore, noncardiologists are often unaware of the specifics and potential complications associated with this procedure. Therefore, PVS is often misdiagnosed as pneumonia, asthma, pulmonary embolism, lung cancer, or other respiratory disorders, and patients frequently undergo unnecessary procedures, including bronchoscopy, pleurocentesis, inferior vena cava filter placement, and lung resection.^10^ In our case, hemoptysis was incorrectly attributed to pneumonia, and transcatheter bronchial artery embolization was performed twice. It is important to improve awareness regarding RAAF-induced PVS among clinicians to facilitate timely referral of these patients to an interventional cardiologist for accurate diagnosis to avoid unnecessary medical interventions and ensure optimal management.

Most patients with PVS present with dyspnea, recurrent cough, expectoration, chest pain, flu-like symptoms and hemoptysis. Recurrent hemoptysis was the predominant symptom in our patient, and although hemoptysis is not rare in patients with PVS, hemoptysis associated with RAAF-induced PVS is relatively rare when considering the overall common causes of hemoptysis. The pathophysiological mechanism contributing to hemoptysis in patients with PVS remains unclear. We concluded that hemoptysis was attributable to increased pulmonary vascular resistance secondary to PVS. Magnetic resonance imaging and pulmonary vein CT angiography are valuable noninvasive diagnostic tools for PVS. Both modalities provide useful information regarding the site and extent of PVS, but the low spatial resolution associated with these modalities is a limitation; therefore, cardiac catheterization and pulmonary artery angiography remain the gold standard for the diagnosis of PVS.

Transcatheter bronchial artery embolization is widely used as a safe and effective treatment to prevent hemoptysis; however, it is ineffective in patients with RAAF-induced PVS because these patients develop hemoptysis secondary to elevated pulmonary vein pressure and reduced venous backflow. Therefore, treatment in these patients is primarily aimed at relieving the obstruction and increasing blood flow through the pulmonary veins. The main treatment modalities for PVS include balloon angioplasty and/or stent implantation. Both methods are known to successfully alleviate symptoms; however, high rates of restenosis have been observed over the long-term follow-up. Compared with balloon dilation, stent implantation is associated with a relatively low restenosis rate, particularly with the use of stents measuring >10 ​mm in diameter. Long-term anticoagulant therapy is necessary after the procedure to avoid pulmonary vein thrombosis. A recent study has reported the use of drug-eluting stents to reduce the restenosis rate,^11^ and excellent stent patency rates were observed in these cases. Further studies including large controlled trials are warranted to conclusively establish the efficacy of these devices. Surgical lobectomy is a life-saving procedure for patients with complete pulmonary vein occlusion and massive hemoptysis. Other options include sutureless venoplasty and pericardial patchplasty.^12^

In conclusion, PVS is a complication of RAAF that is often misdiagnosed owing to lack of adequate awareness regarding this condition among noncardiologists. Unexplained massive hemoptysis should alert clinicians regarding the possibility of RAAF-induced PVS in patients with such a clinical presentation. Pulmonary vein CT angiography is a valuable noninvasive method to diagnose PVS. Minimally invasive treatments, such as balloon dilation and pulmonary vein stenting have been used to treat PVS; however, their efficacy remains controversial owing to the relatively high recurrence rates associated with these interventions.

## Patient consent

Written informed consent was obtained from patients for publication of this case reports and any accompanying images.

## Declaration of interests

The authors declare that they have no known competing financial interests or personal relationships that could have appeared to influence the work reported in this paper.
